# Mr. Ricards, on Hydrocephalus

**Published:** 1801-04

**Authors:** J. Ricards

**Affiliations:** Member of the Royal College of Surgeons, London. Brentford


### *To the Editors of the Medical and Phyſical Journal.*

Gentlemen,

### *To the Editors of the Medical and Phyſical Journal.*

Gentlemen,

I Obſerve in Number xxiv. of the Medical and Phyſical Journal, a caſe of Apoplexia Hydrocephalica, related by Dr. Garnett, wherein the ſymptoms and progreſs of the diſeaſe are related with equal perſpicuity and exactneſs, giving an opportunity for many very valuable remarks. I am convinced of the great great utility which refults from the publication of the fuccefsful cafes of thofe difeafes, which are rather unfrequent, and moft generally fatal, which induces me to prefent the following ideas, deduced from a minute attention to the annexed cafe.

Mafter L——, aged about 8 years, the fon of a medical gentleman of judgment and ability in his profeffion, a child of a remarkably active, volatile difpofition, and acute underftanding for his years, became fomewhat indifpofed about the latter part of October, or the beginning of November, 1800, being then at a confiderable diftance from home; at which time he was under the care of a medical gentleman in the country, but continued getting gradually worfe till the laft week in November, when he was brought home. His father, at firft view of him, was very much alarmed, for his appearance was fuch as to give him ferious apprehenfion with regard to his prefent fafety: his debility was fo great, that he could not fupport himfelf on his feet, and fymptoms were fo extremely urgent, that it was not expected that he could furvive through the night. From the affemblage of fymptoms, it appeared to his father, that his difeafe was hydrocephalus; he therefore immediately gave him a tolerably large dofe of jalap and calomel, applied a blifter to the nape of the neck, and the following day began the adminiftration of calomel, in union with fquills; and, on account of the extreme debility, as much of the cinchona in fubftance as the ftomach could bear.

But as the fymptoms continued of a very difagreeable nature, and not chufing to rely folely on his own opinion in fuch a cafe, he requefted me to meet himfelf and another medical gentleman, which I accordingly did, on the 4th of Dec. 1800, a few days after his return home.

On examining into his fenfations, we found that he referred all his difeafe to his head, which he faid was in almoft conftant pain, particularly on the vertex, which was confiderably increafed by preffure; but although the pain was conftant in fome degree, yet it was not equally fo, for feveral times in the day it became fo much more violent, as to deprive him of fenfe for the time: He would frequently fhriek out in his fleep; awake fuddenly; and if not prevented, ftart from his bed. He appeared to have loft all his activity, and to be funk in a kind of ftupor, particularly on the exacerbation of pain. The pupils of his eyes were dilated, and the iris appeared very infenfible, contracting very little on fudden expofure to light, which however, he feemed to wifh to avoid as much as poffible, by turning his face from the window or candle. A degree of ftrabifmus exifted, being very confiderable when the pain was the greateft, at which time his fight was very imperfect.

His

His eyes had loſt their power of directing him with exactneſs to any object; for if he was deſired to touch any thing at arm's length, he could not directly guide his hand to it, but always conſiderably to one ſide. He complained of pain on any ſudden motion, eſpecially of his head, which he always moved very ſlow; nor could he turn it round, without at the ſame time moving his body.

His pulſe was ſlow, ſmall, and remarkably irregular.

His bowels were very much inclined to coſtiveneſs, being very little acted on by cathartics of a powerful kind.

He had frequent fluſhings, and often ſickneſs of the ſtomach and vomiting.

He had leſs debility than at his return home, which ſeems to be owing to his having taken very freely of the bark.

The quantity of urine has been increaſed by the exhibition of the ſquills.

On contemplating theſe ſymptoms, it appeared to us that they aroſe from preſſure of ſome kind upon the brain; and as there had been no known blow, or other external injury, and being no appearance of any, we concluded that the diſeaſe Hydrocephalus exiſted; and under this idea our mode of treatment was conducted.

It was agreed, therefore, that the head ſhould be ſhaved, and a bliſter applied over the whole ſurface of the ſcalp, and kept open for a conſiderable time.

That he ſhould continue taking the bark in the ſame form as he had begun it, which was as follows.

℞. Pulv. cort. cinchon. flav. ʒj. Tinct. gentian. c. ʒß. Aq. diſtill. ℥iijß. M. Cap. coch. ij. ampl. 4 q. horâ.

And every other morning the following powder.

℞. Calomel præp. Gum. Gambog. pulv. aa. gr. iv. M. ft. pulv.

That he ſhould take a moderate quantity of red wine daily.

Our intention in the powder was to produce a very briſk cathartic effect; in which, however, we were at firſt diſappointed, as this doſe had no viſible effect; it was gradually increaſed till he took of calomel and gamboge each eight grains, which quantity, after remaining rather more than half an hour upon his ſtomach, cauſed gentle vomiting, and only one or two ſlight alvine evacuations; the ſame effect reſulted from the addition, at one time, of ten grains of pulv. jalap to the above doſe; and at another time of aloes ſocot. gr. xv. But wiſhing to produce a ſtill greater degree of action on the inteſtines, our purpoſe was at length very effectually anſwered by the following.

℞. Calomel præp. Gum. gamb. pulv. aa. gr. viij. Elaterii pulv. gr. ij. M. ft. Pulv. altern. auror. ſum.

This

This produced very brifk evacuations at each time of repeating it; after each repetition, however, he appeared better and more lively. The plan was continued for feveral weeks, during which every fymptom of the difeafe gradually fubfided, until his priftine ftate of health was completely renewed.

In the paper before alluded to, by Dr. Garnett, much doubt feems to be entertained, whether fuch a difeafe as the term Hydrocephalus in reality imports, ever exifts, except as a confequence of another difeafe whofe proximate caufe is an increafed plenitude of the veffels of the brain, which the Doctor thinks is alone fufficient to produce all the fymptoms which are definitive of the Apoplexia Hydrocephalica of Cullen. If fuch indeed were the cafe, the ftrict antiphlogiftic treatment would certainly be that from which moft fuccefs could be expected. In the firft inftance, very probably, the difeafe may confift of increafed determination to, and fulnefs of, thefe veffels; but in the above cafe, I am really of opinion, whatever might have been the ftate of the parts at the firft attack, that at the time the above plan was prefcribed, effufion had actually taken place, as fuch plan was not altogether adapted for the relief of fymptoms merely arifing from increafed plethora.

Under the idea, then, that effufion of ferum has taken place, the indications of cure certainly muft be to excite an increafed action of the abforbent veffels, at the fame time we give tone and vigour to the general fyftem, as in other cafes of dropfical accumulation. Much has been faid of the ufe of mercury in thefe cafes; given, not as a cathartic, but in fuch a way as to excite what may be called the mercurial action in the fyftem; and fome phyficians, of undoubted eminence in their profeffion, adminifter it in fuch a manner as to produce copious ptyalifm as fpeedily as poffible. It is certain, indeed, that mercury is a powerful promoter of abforption, yet the debility it occafions is frequently extreme, which is a circumftance much to be avoided in this difeafe; we even find, that by the continued ufe of mercury, either the action of the exhalents is fo much increafed, or that of the abforbents fo much decreafed, that dropfical accumulations are produced; as we not unfrequently find that anafarcous fwellings are the confequences of a mercurial courfe: For, probably, although the mercury at firft excites the abforbents to a more energetic action, yet, after a time, thefe veffels, being governed by the fame laws which influence all moving fibres, fuffer increafed debility as the confequence of this excefs of action.

If we fuppofe that no dropfical accumulation has taken place, but merely increafed determination to the head, can we expect advantage from mercury, as being a powerful ftimulant lant, and increafing very much the action of the arterial fyftem? To obviate thefe effects, Dr. Garnett, in the cafe above alluded to, unites it with Digitalis, a medicine well known as the moft powerful of all fedatives.* There is no doubt that many cafes have been treated by the mercurial plan with fuccefs; but feldom, I believe, has mercury been accompanied with cathartics, blifters, and other remedies.

Another reafon why we omitted the mercurial treatment in this cafe was, that in the courfe of the laft year one gentleman affured me that he had had opportunities of opening no lefs than fix heads of patients who had died with the fymptoms of this difeafe, in all of whom the mercurial treatment had been carried to its full extent, and in all there was a confiderable effufion of ferum in the ventricles.

Therefore it appears to me, that draftic purgatives, frequently adminiftered, have a much fairer chance of fuccefs by increafing very powerfully the action of the abforbents, while they do not produce that debility of the fyftem, which is the confequence of mercury; at the fame time that the ftrength is prevented from failing, or the priftine vigour re-eftablifhed, if it has already begun to fail, by the adminiftration of the cinchona in fuch dofes as the ftomach will retain; not, however, omiting what I conceive to be the moft powerful of all the remedies employed, a blifter to the head, to be kept difcharging for a length of time.

Whether this mode of treatment, which has fo happily fucceeded in the prefent cafe, will be equally fuccefsful in others, or whether it will prove effectual in as large, or a ftill larger proportion than thofe which terminate to our wifhes by the mercurial plan, experience can alone determine. The cafe, as it is, I have impartially related; and I think, that every fuccefsful cafe of a difeafe fo frequently fatal as the Apoplexia Hydrocephalica, fhould be made known. I am, &c.

J. RICARDS,
Member of the Royal College of Surgeons, London.

*Brentford, Feb.* 16, 1801.

---

* Quere, Does not the good effects of mercury depend on the acceleration it produces in the circulation? And would not digitalis, or other powerful fedatives, prevent thofe good effects in the treatment of Lues? &c.

